# Effects of eradication of *Helicobacter pylori* on oral malodor and the oral environment: a single-center observational study

**DOI:** 10.1186/s13104-020-05253-5

**Published:** 2020-08-28

**Authors:** Nao Suzuki, Richiko Beppu, Masahiro Yoneda, Toru Takeshita, Mikari Asakawa, Yoshihisa Yamashita, Takashi Hanioka, Takao Hirofuji, Tetsuo Shinohara

**Affiliations:** 1 Department of Preventive and Public Health Science, Fukuoka Dental College, Fukuoka, Japan; 2 Oral Medicine Research Center, Fukuoka Dental College, Fukuoka, Japan; 3 Department of General Medicine, Fukuoka Dental College, Fukuoka, Japan; 4 Department of General Dentistry, Fukuoka Dental College, Fukuoka, Japan; 5 Section of Preventive and Public Health Dentistry, Faculty of Dental Science, Kyushu University, Fukuoka, Japan; 6 OBT Research Center, Faculty of Dental Science, Kyushu University, Fukuoka, Japan; 7 Present Address: Department of Proctology, Fukuseikai Hospital, Fukuoka, Japan; 8 Present Address: Department of Surgery, Fukuseikai Hospital, Fukuoka, Japan

**Keywords:** Antimicrobial therapy, *Helicobacter pylori*, Oral bacteria, Oral malodor, Organoleptic test, Volatile sulfur compound

## Abstract

**Objective:**

Although a relationship between *Helicobacter pylori* and oral malodor has been suggested, it remains to be confirmed. One reason for this is that many studies assess oral malodor subjectively. Another reason for the uncertainty is that the reduction in oral malodor may be due to the effect of antibiotics on the oral microbiota. In this study, changes in oral malodor along with the eradication treatment of *H. pylori* were investigated by organoleptic test and gas chromatography. In addition, the salivary bacterial composition and clinical parameters were analyzed.

**Results:**

The organoleptic test score, hydrogen sulfide and dimethyl sulfide concentrations, and all clinical parameters except for tongue-coating score were significantly decreased at 1 week compared with baseline. Although antibiotic treatment also altered the overall composition of the salivary bacterial population, it had recovered at 7 weeks. On the date that *H. pylori* was determined to have been eradicated from all of the subjects (7 weeks after treatment), only the organoleptic test score was significantly lower compared with baseline. The hydrogen sulfide and dimethyl sulfide concentrations were non-significantly lower than those at baseline.

## Introduction

A relationship between *Helicobacter pylori* and oral malodor has been known since the identification of *H. pylori* [1]. Upper gastrointestinal disorders can cause oral malodor, but oral malodor is linked to the oral cavity in 90% of cases [2]. Several cross-sectional studies have reported that *H. pylori*-infected individuals had oral malodor [3–5], but no difference in exhaled breath or stomach air [5] compared to uninfected individuals. Therefore, it has often been hypothesized that gastric or oral *H. pylori* infection alters oral conditions, possibly leading to oral malodor [6–8].

Observational studies of the effect of *H. pylori* eradication on oral malodor would help better to clarify the relationship between oral malodor and *H. pylori* infection [9–12]. Such studies should consider two points. First, it is necessary to evaluate oral malodor objectively. Prior studies assessed subjective symptoms of oral malodor using questionnaires and interviews; however, it is difficult for an individual to evaluate their own oral malodor accurately [13, 14]. Second, it is necessary to clarify the effects of the antibiotics used for *H. pylori* eradication on the oral microbiota, since those antibiotics are reportedly effective against periodontal disease [15, 16].

In this study, we objectively evaluated oral malodor changes along with the eradication treatment of *H. pylori* by measuring the concentrations of volatile sulfur compounds (VSCs) by gas chromatography (GC) and organoleptic test (OLT) as the primary outcome. The secondary outcomes included oral symptoms and bacterial parameters. In addition, 16S rRNA gene sequencing was performed to evaluate the direct effect of the antibiotics used to eradicate *H. pylori* on the oral microbiota.

## Main text

### Materials and methods

#### Study design

An observational study was conducted between September 2014 and May 2016. The study population comprised patients who presented with gastric symptoms and were diagnosed with *H. pylori* infection based on positive rapid urease tests conducted on gastric biopsy specimens. The inclusion criteria were as follows: 20 years or older, having own teeth, and no oral symptoms that require immediate treatment. Those with drug allergies and pregnant women were excluded. Twelve patients were analyzed (Additional file 1; Table S1). They were prescribed a 1-week course of a triple-drug regimen (30 mg lansoprazole, 750 mg amoxicillin, and 200 mg clarithromycin, twice daily) as the primary eradication regimen. In cases in which the primary treatment failed, 250 mg metronidazole was used instead of clarithromycin as the secondary regimen.

#### Clinical examinations

Oral malodor and oral clinical symptoms were evaluated on the date of initiation of *H. pylori* eradication treatment and 1 and 7 weeks thereafter. The severity of oral malodor was determined by OLT [13] and GC. A gas chromatograph (GC2014; Shimadzu, Kyoto, Japan) was used to assay the concentrations of H_2_S, CH_3_SH, and CH_3_SCH_3_ in mouth air. The average probing pocket depth (PPD), the percentage of bleeding on probing (BOP) values, the plaque index (PlI) [17], and the tongue coating score (TCS) [14] were evaluated. The ammonia concentration was measured using a portable ammonia-monitoring device (ATTAIN; Taiyo, Osaka, Japan). Saliva samples were obtained by stimulation using chewing gum (CheckBuf; Morita, Osaka, Japan) and subjected to sequencing of the 16S rRNA gene amplicons and quantitative analysis of bacterial species. These examinations were conducted at 9 am; the subjects were prohibited from eating, drinking, chewing, smoking, brushing teeth, or rinsing their mouth after waking up.

#### Quantitative analysis of bacterial species in saliva

Quantitative analysis of 12 bacterial species and all bacteria in saliva was performed using the fibrous DNA chip Genopal^®^ (Mitsubishi Chemical, Tokyo, Japan). The bacterial species were classified into the red (*Porphyromonas gingivalis*, *Tannerella forsythia*, and *Treponema denticola*), orange (*Campylobacter rectus*, *Fusobacterium nucleatum*, *Prevotella intermedia*, and *Prevotella nigrescens*), green (*Aggregatibacter actinomycetemcomitans* and *Capnocytophaga gingivalis*), and yellow (*Streptococcus gordonii*, *Streptococcus intermedius*, and *Streptococcus mutans*) groups [18], and the proportions of these groups among the total oral bacteria were evaluated.

#### Sequencing of 16S rRNA gene amplicons

DNA was extracted from each saliva sample [19] and the V1–V2 regions of the 16S rRNA gene were amplified by PCR using the universal bacterial primers 8F and 338R with adaptor and sample-specific 8-base tag sequences [20]. Following emulsion PCR, sequencing was performed on the Ion PGM using an Ion PGM Hi-Q View sequencing kit (Thermo Fisher Scientific, Waltham, MA). The raw sequencing reads were quality-filtered [21]. The quality-checked reads were assigned to the appropriate sample by examining the tag sequence. Similar sequences were assigned to operational taxonomic units (OTUs) using UPARSE [22], with a minimum pairwise identity of 97%. The taxonomy of each representative sequence was determined using blast against oral bacterial 16S rRNA gene sequences in the Human Oral Microbiome Database [23]. Nearest-neighbor species with ≥ 98.5% identity were selected as candidates for each OTU. The taxonomy of an OTU without any hits was determined to the genus level using the Ribosomal Database Project classifier with a minimum support threshold of 80%. The alpha diversity index and UniFrac distances [24] were calculated following rarefaction to 5000 reads per sample.

#### Statistical analysis

The Wilcoxon signed-rank test was used to compare malodor, clinical, and bacterial parameters among baseline, 1 week, and 7 weeks. The relative abundance of bacterial genera after 1 and 7 weeks were compared with those at baseline using Dunnett’s test. Statistical analyses were conducted using R software, version 3.6.1 [25].

## Results

### Oral malodor and clinical parameters

Table 1 shows the changes of the oral malodor and clinical parameters. The values of all parameters decreased at 1 week compared with baseline, and the decreases in the OLT score, H_2_S and CH_3_SCH_3_ concentrations, PPD, BOP, PlI, and ammonia concentration were significant. On the date when *H. pylori* had been eradicated from all subjects, the values of all parameters other than the CH_3_SH concentration were lower than those at baseline but tended to be higher than those at 1 week. Only the OLT score was significantly lower than that at baseline (*P *= 0.037). The comparisons between the success and failure groups after primary eradication treatment showed that the OLT score, total VSC concentration, BOP, and PPD in the failure group were increased slightly at 7 weeks, as compared with 1 week, but were unchanged in the success group (Additional file 2: Figure S1).

**Table 1:** Clinical parameters at baseline, the date of treatment completion, and the date of eradication of *H. pylori*

Parameter	Baseline	Date of treatment completion	Determination of date of successful eradication
OLT score	2.25 [1.50, 3.00]	1.50 [0.89, 1.56]*	1.75 [1.19, 2.00]*
VSCs (ng/10 mL)	2.29 [1.26, 6.72]	1.69 [0.25, 2.68]	1.57 [0.78, 5.23]
H_2_S (ng/10 mL)	1.19 [0.50, 4.88]	0.51 [0.13, 1.75]*	0.56 [0.35, 3.14]
CH_3_SH (ng/10 mL)	0.55 [0.36, 1.24]	0.29 [0.09, 0.95]	0.58 [0.21, 1.35]
CH_3_SCH_3_ (ng/10 mL)	0.60 [0.38, 0.70]	0 [0.00, 0.43]*	0.32 [0.24, 0.55]
PPD (mm)	3.02 [2.98, 3.24]	3.0 [2.92, 3.02]*	3.0 [2.93, 3.02]
BOP (%)	7.64 [2.98, 21.0]	4.27 [1.74, 6.33]*	4.74 [2.22, 14.0]
PlI	0.67 [0.21. 0.83]	0.29 [0.21, 0.39]*	0.65 [0.35, 0.84]
TCS	2.00 [1.75, 2.00]	1.00 [1.00, 2.00]	1.50 [1.00, 2.00]
Ammonia (ppm)	39.0 [20.8, 45.8]	14.0 [0.00, 18.8]*	30.0 [20.0, 50.0]

Median [IQR]

PlI, plaque index; TCS, tongue-coating score; * *P* < 0.05 vs. baseline by Wilcoxon signed-rank test

### Quantitative analysis of bacterial species in saliva

The total bacterial amounts (median [IQR], log copies/mL) in saliva at baseline, 1 week, and 7 weeks with the primary eradication regimen were 7.7 [7.4–8.0], 7.4 [7.2–8.0], and 7.9 [7.5–8.1], respectively. The proportions of bacterial groups were compared in the success and failure groups with the primary eradication regimen (Table 2). The proportion of the red group was lower at 7 weeks than at baseline in both groups, although the difference was significant only in the success group (*P* = 0.039). In addition, the proportions of *T. forsythia* (*P* = 0.098) and *T. denticola* (*P* = 0.098) were decreased at 7 weeks compared with baseline and at 1 week, respectively, albeit not significantly so (Additional file 3: Table S2). The proportions of *C. rectus*, *F. nucleatum* and *P. nigrescens* at baseline were non-significantly higher in the failure group than in the success group.

**Table 2:** Bacterial groups in saliva by the primary eradication regimen outcome

Bacterial group	Eradication	Baseline	1 week	7 weeks
Red group	Success	0.04 [0.03, 0.07]	0.02 [0.01, 0.04]	0.03 [0.02 0.03]*
	Failure	0.05 [0.04, 0.05]	0.03 [0.02, 0.06]	0.02 [0.02, 0.02]
Orange group	Success	2.14 [1.40, 5.56]	1.01 [0.12, 4.38]	3.29 [2.62, 7.17]
	Failure	2.63 [2.11, 4.47]	0.78 [0.43, 3.87]	6.17 [5.0, 6.65]
Green group	Success	0.05 [0.01, 0.28]	0.22 [0.01, 0.77]	0.03 [0.0, 0.09]
	Failure	0.05 [0.04, 0.17]	0.35 [0.18, 0.45]	0.02 [0.01, 0.32]
Yellow group	Success	0.55 [0.42, 1.17]	0.28 [0.15, 0.61]	0.75 [0.38, 0.89]
	Failure	0.80 [0.62, 1.08]	1.05 [0.78, 2.24]	0.38 [0.36, 0.67]

Median [IQR]

* *P* < 0.05 vs. baseline by Wilcoxon signed-rank test

1 week, date of treatment completion. 7 weeks, determination of date of eradication

### Bacterial diversity and composition in saliva

**Fig. 1:**
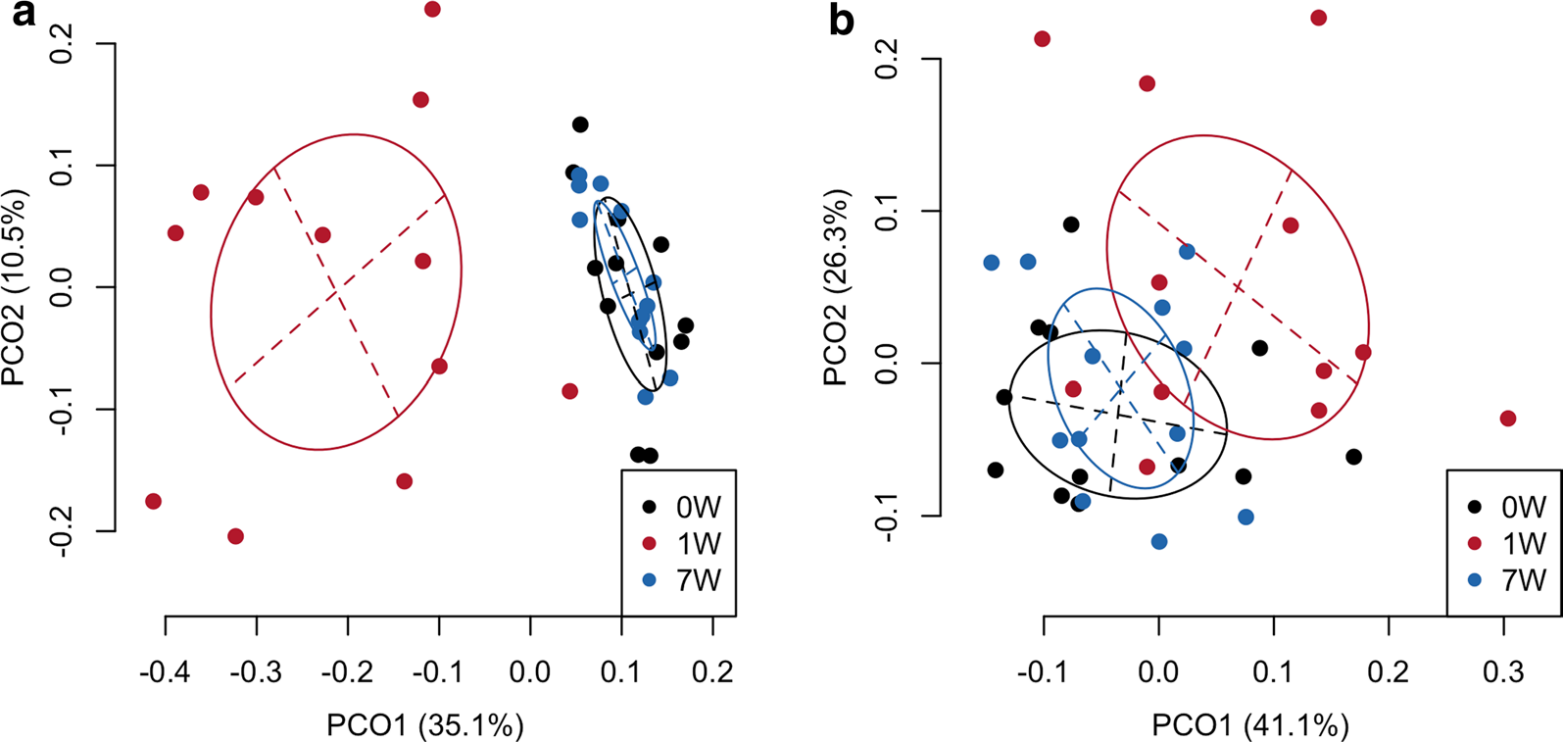
A principal coordinate analysis (PCoA) plot showing the similarity relationship between saliva samples from 12 patients at three phases of treatment using unweighted (**a**) and weighted (**b**) UniFrac distance matrices with the primary eradication regimen. 0 W, baseline; 1 W, date of treatment completion (1 week); 7 W, determination of date of *H. pylori* eradication (7 weeks). The ellipse covers 67% of the samples belonging to each phase group

The alpha diversity index was decreased at 1 week compared with baseline in every individual and increased to the baseline level again on the determination of the date of *H. pylori* eradication by the primary regimen (Additional file 4: Figure S2). A UniFrac analysis demonstrated that antibiotic treatment for *H. pylori* infection altered the overall composition of the salivary bacterial populations, although they had recovered on the determination day of eradication (Fig. 1). The relative abundance of *Rothia* was significantly lower in the saliva at 1 week compared with baseline (*P *= 0.005), whereas no significant difference in the relative abundance of the genus was observed between baseline and the date of determining *H. pylori* eradication by the primary regimen (Additional file 5: Figure S3).

## Discussion

A recent systematic review concluded that oral malodor is reduced by eradication of *H. pylori* [26]. However, most of the studies included in that review assessed subjective symptoms of oral malodor using a questionnaire [11, 12, 27, 28]. Few studies have assessed oral malodor using a portable sulfide monitor or OLT [10, 29]. Oral malodor is influenced by psychological factors, and it is difficult for individuals to evaluate their own oral malodor accurately [13, 14]. To elucidate the relationship between oral malodor and *H. pylori* infection, objective evaluations of oral malodor are needed, such as measuring odor components and OLT performed by experts. This is the first study to measure VSC concentrations by GC and to perform the OLT to evaluate the effect of *H. pylori* eradication on oral malodor objectively. As a result, the OLT score was significantly decreased after *H. pylori* eradication compared with baseline. In contrast, the concentrations of H_2_S and CH_3_SCH_3_ were decreased on the date of eradication compared with baseline, albeit not significantly so. The amelioration of oral malodor was not due to a direct effect of antibiotics on oral bacteria, because the bacterial composition was not different from that before eradication and the total oral bacterial count was not significantly different between after *H. pylori* eradication and baseline. Use of antibiotics in combination with mechanical therapy for periodontal disease resulted in significant differences in the richness and dissimilarity of the oral microbiota after 2 months, whereas the evenness and diversity were not affected [30]. It is possible that the effect on the oral microbiota differs between antibiotics alone and antibiotics plus mechanical therapy. Concerning the significant reduction of the OLT score, ammonia, methylamine, dimethylamine, propionic acid, butyric acid, indole, skatole, and cadaverine may cause oral malodor in addition to VSCs [31]. More studies on the involvement of substances other than VSCs in oral malodor associated with *H. pylori* are needed.

After initiating the primary eradication regimen, there were differences in the clinical findings and the proportions of some bacterial species between the success and failure groups. We postulate that these differences are key to successful eradication. With the primary eradiation regimen, the OLT score, total VSC concentration, BOP, and PPD tended to increase slightly, after decreasing at 1 week, in the failure group, but these parameters remained low in the success group. The success group showed a significant decrease in the proportion of red complex species (*P* = 0.039). At 1 week, the proportions of *S. gordonii* and *C. gingivalis* were increased and decreased in the failure and success groups, respectively. The proportions of *C. rectus*, *F. nucleatum*, and *P. nigrescens* at baseline were higher in the failure group than in the success group. Periodontopathic bacteria such as *F. nucleatum* and *P. nigrescens* produce indole, skatole, and acetic, propionic, and butyric acids [32, 33]. Compared with the success group, the higher proportions of these bacteria in the failure group may be related to the higher OLT score at baseline, despite the lower VSC and PPD levels. *C. rectus* and *H. pylori* share several antigens that may play a role in the initiation and progression of periodontal disease [34, 35]. A high proportion of *C. rectus* may indicate active periodontal or gastric infection.

The ability of *H. pylori* to produce H_2_S and CH_3_SH is strain-specific [36]. *H. pylori* has been detected in oral specimens [6, 7, 37], and its prevalence was associated with the progression of periodontitis. There is disagreement as to whether *H. pylori* is present in the oral cavity. The bacterial communities in the oral cavity are very complex, with over 500 species, and include many VSC-producing bacteria [2]. The oral malodor associated with *H. pylori* is unlikely to be caused by its production of VSCs, but rather by altering the oral environment and the populations of some oral bacteria. Chronic systemic inflammation and periodontitis interact via the systemic inflammatory response [38]. Thus, irrespective of the presence or absence of *H. pylori* infection in the oral cavity, gastric inflammation by *H. pylori* may exacerbate oral malodor via its effect on the oral environment.

## Limitations

The current study included a small number of subjects.

## Supplementary information

**Additional file 1: Table S1.** Profile of the study population.

**Additional file 2: Figure S1**. Oral malodor and clinical parameters in the *H. pylori* eradication success and failure groups after the primary eradication regimen.

**Additional file 3: Table S2.** Proportions of bacterial species in saliva by the primary eradication regimen outcome. Median [IQR].

**Additional file 4: Figure S2.** Changes in two alpha diversity indices, the number of OTUs (A) and the Shannon diversity index (B), in saliva after the primary eradication regimen. 0W, baseline; 1W, date of treatment completion (1 week); 7W, determination of the date of *H. pylori* eradication (7 weeks).

**Additional file 5: Figure S3.** Relative abundance of the predominant bacterial genera in saliva after the primary eradication regimen. Only genera with a mean relative abundance of ≥ 1% are shown. 0W, baseline; 1W, date of treatment completion (1 week); 7W, determination of the date of *H. pylori* eradication (7 weeks). **P < 0.01, Dunnett’s test (*vs.* baseline).

## Data Availability

The datasets used and analyzed during the current study are available from the corresponding author on reasonable request.

## Abbreviations

BOP: Breeding on probing
GC: Gas chromatography
IQR: Interquartile range
PlI: Plaque index
OLT: Organoleptic test
OTU: Operational taxonomic unit
PPD: Probing pocket depth
TCS: Tongue coating score
VSC: Volatile sulfur compound
