# Supplementary material for: Effects of eradication of Helicobacter pylori on oral malodor and the oral environment: a single-center observational study
Source: BMC Res Notes. 2020 Aug 28;13:406. doi: 10.1186/s13104-020-05253-5 (PMC7455998; doi:10.1186/s13104-020-05253-5)
Supplement: Supplementary file 1 — Additional file 1: Table S1. Profile of the study population. [file 13104_2020_5253_MOESM1_ESM.docx]

**Additional Files**

Additional file 1: Table S1. Profile of the study population.

| Case | Age (years) | Primary eradication regimen | Secondary eradication regimen |
| --- | --- | --- | --- |
| 1 | 49 | S† |  |
| 2 | 57 | F‡ | S |
| 3 | 66 | S |  |
| 4 | 21 | F | S |
| 5 | 62 | S |  |
| 6 | 73 | S |  |
| 7 | 65 | S |  |
| 8 | 66 | S |  |
| 9 | 72 | S |  |
| 10 | 68 | S |  |
| 11 | 43 | S |  |
| 12 | 72 | F | S |

† Successful eradication of *H. pylori*, ‡ failed eradication of *H. pylori*
