# Supplementary figures and images for: Effects of eradication of Helicobacter pylori on oral malodor and the oral environment: a single-center observational study
Source: BMC Res Notes. 2020 Aug 28;13:406. doi: 10.1186/s13104-020-05253-5 (PMC7455998; doi:10.1186/s13104-020-05253-5)

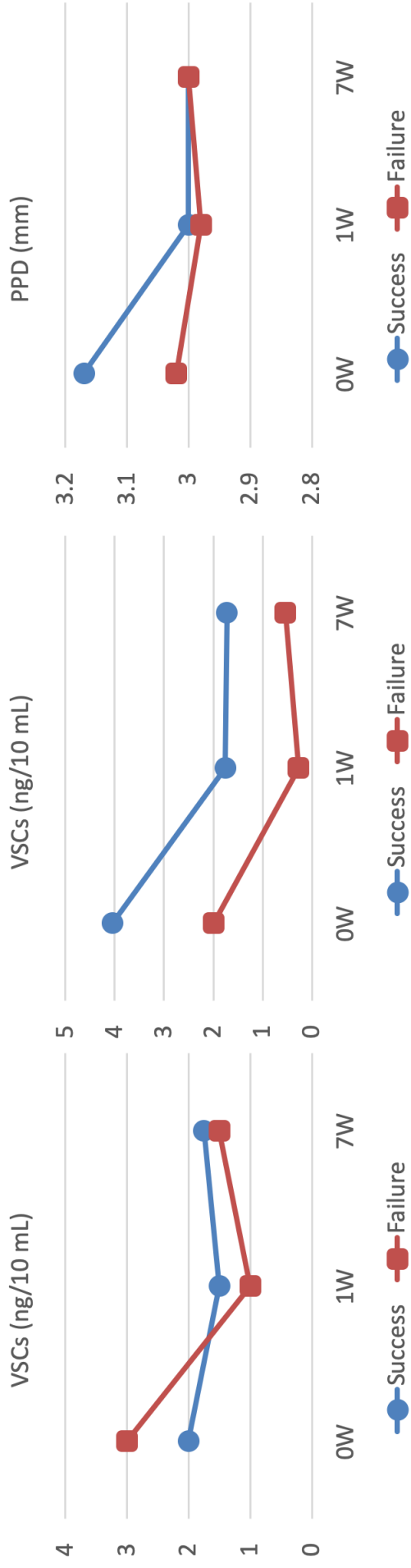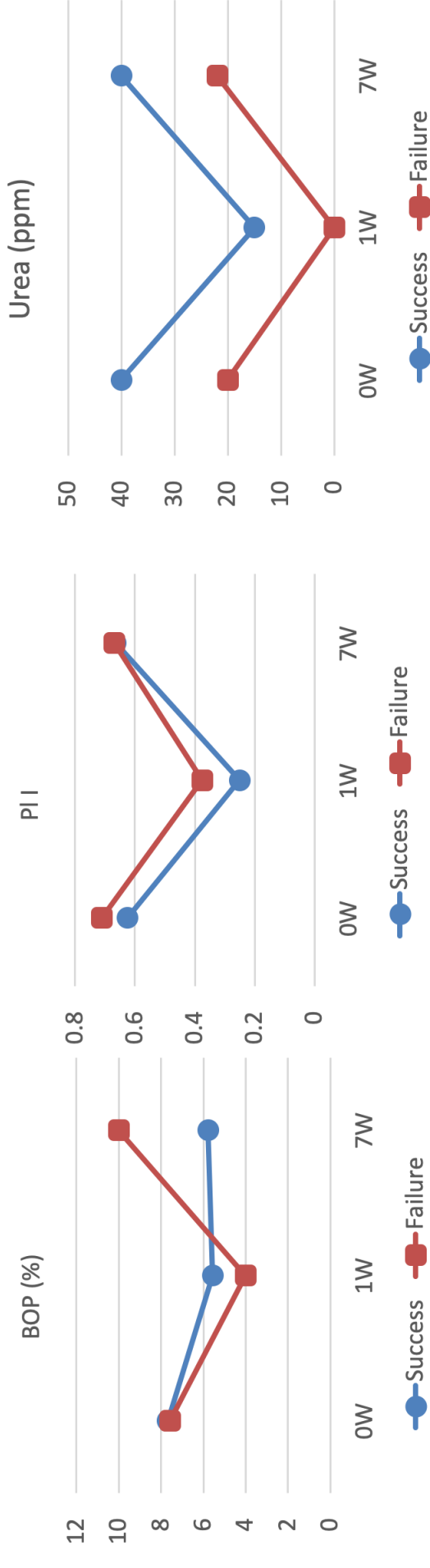

Supplement: Supplementary file 2 — Additional file 2: Figure S1. Oral malodor and clinical parameters in the H. pylori eradication success and failure groups after the primary eradication regimen. [file 13104_2020_5253_MOESM2_ESM.pdf]

Number of identified OTUs

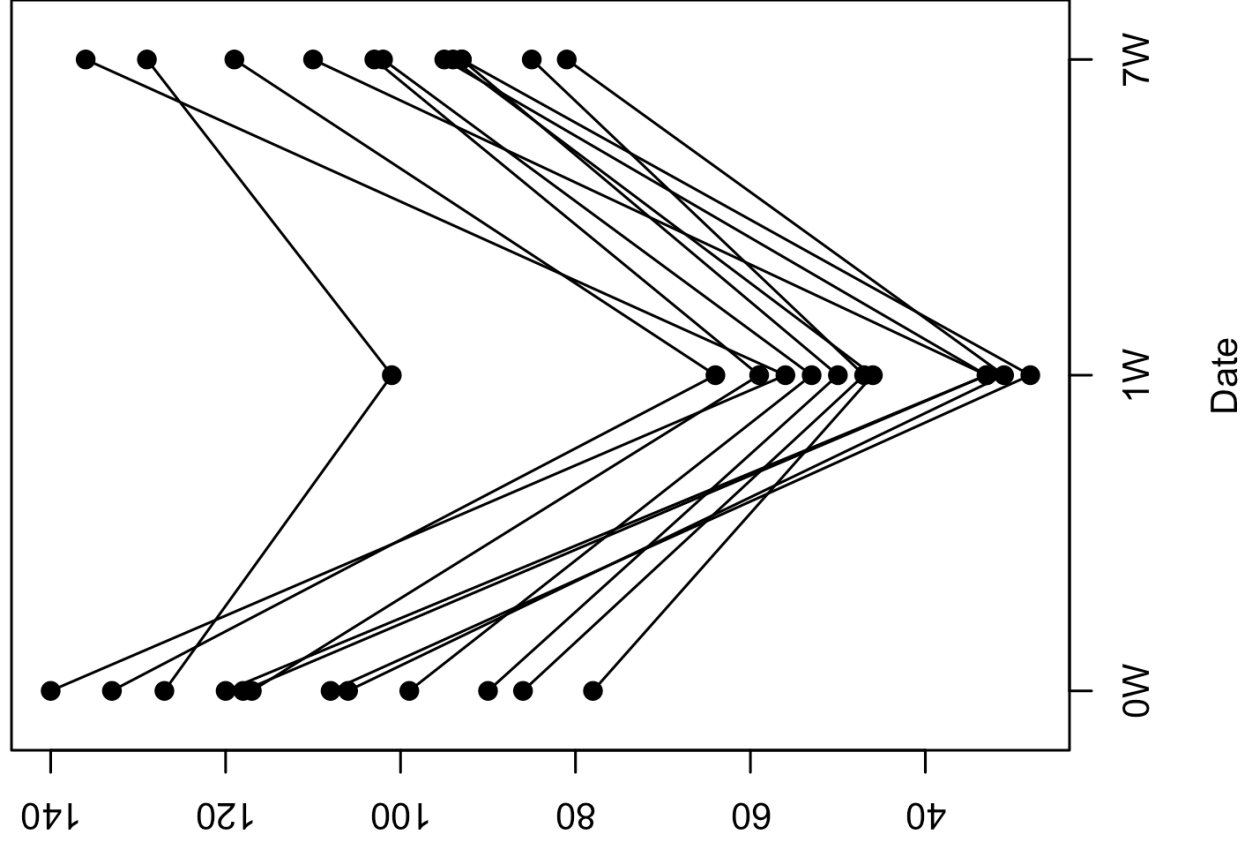

Shannon diversity index

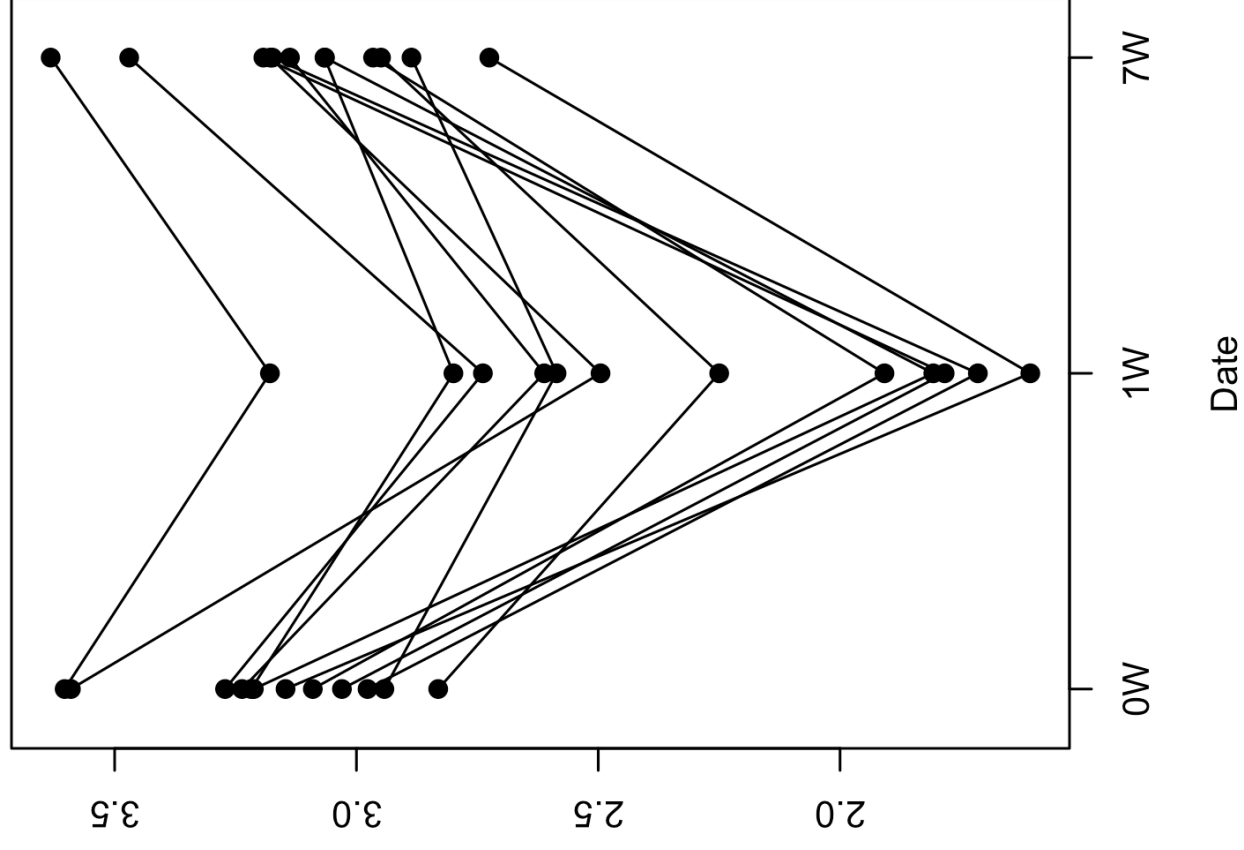

Supplement: Supplementary file 4 — Additional file 4: Figure S2. Changes in two alpha diversity indices, the number of OTUs (A) and the Shannon diversity index (B), in saliva after the primary eradication regimen. 0W, baseline; 1W, date of treatment completion (1 week); 7W, determination of the date of H. pylori eradication (7 weeks). [file 13104_2020_5253_MOESM4_ESM.pdf]

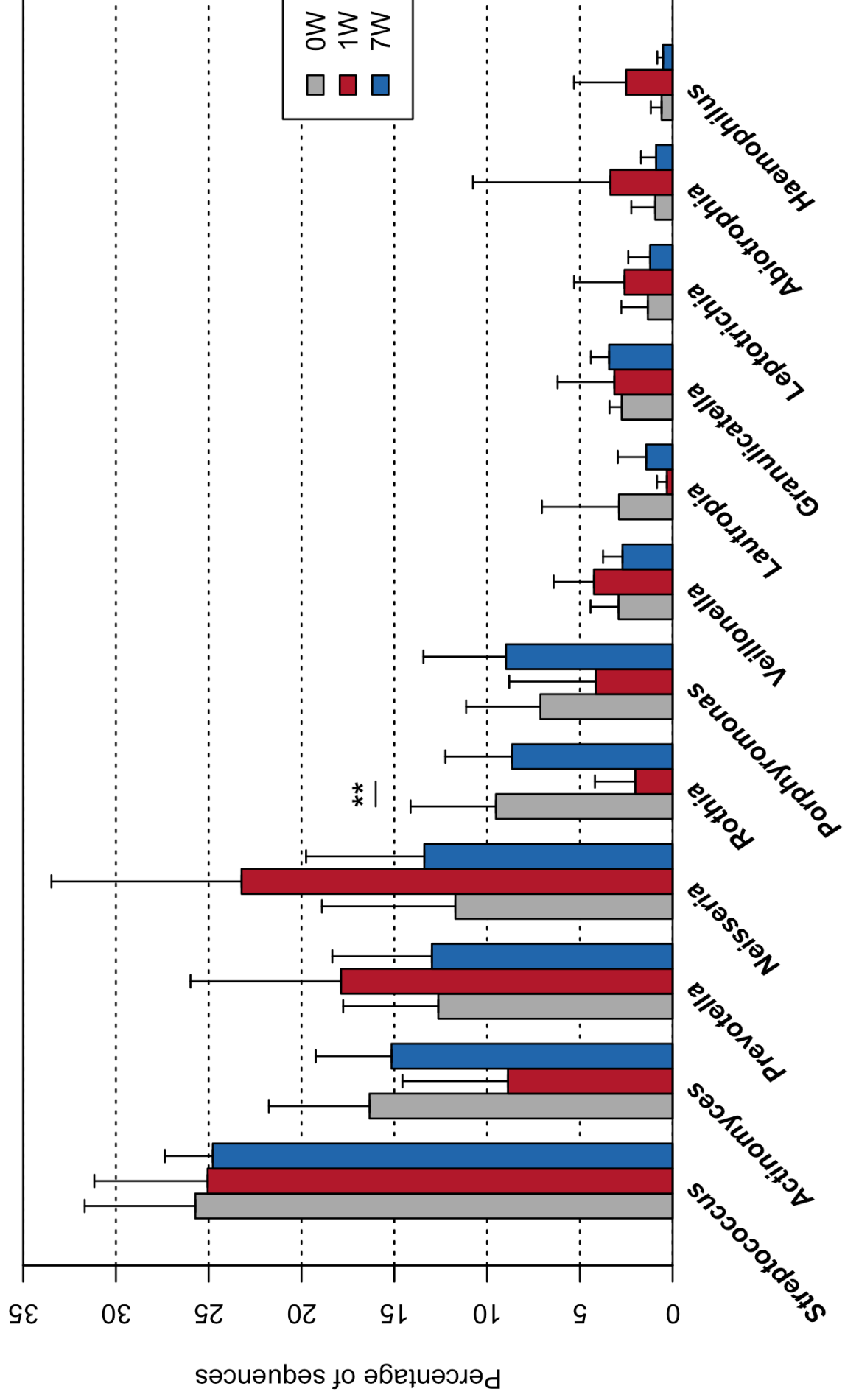

Supplement: Supplementary file 5 — Additional file 5: Figure S3. Relative abundance of the predominant bacterial genera in saliva after the primary eradication regimen. Only genera with a mean relative abundance of ≥ 1% are shown. 0W, baseline; 1W, date of treatment completion (1 week); 7W, determination of the date of H. pylori eradication (7 weeks). **P < 0.01, Dunnett’s test (vs. baseline). [file 13104_2020_5253_MOESM5_ESM.pdf]
