# Supplementary material for: Effects of eradication of Helicobacter pylori on oral malodor and the oral environment: a single-center observational study
Source: BMC Res Notes. 2020 Aug 28;13:406. doi: 10.1186/s13104-020-05253-5 (PMC7455998; doi:10.1186/s13104-020-05253-5)
Supplement: Supplementary file 3 — Additional file 3: Table S2. Proportions of bacterial species in saliva by the primary eradication regimen outcome. Median [IQR]. [file 13104_2020_5253_MOESM3_ESM.docx]

Additional file 3: Table S2. Proportions of bacterial species in saliva by the primary eradication regimen outcome. Median [IQR].

| Bacterial species | Eradication | Baseline | 1 Week | 7 Weeks |
| --- | --- | --- | --- | --- |
| *P. gingivalis* | Success  Failure | 0.01 [0.00, 0.01]  0.00 [0.00, 0.00] | 0.00 [0.00, 0.00]  0.00 [0.00, 0.00] | 0.00 [0.00, 0.01]  0.00 [0.00, 0.00] |
| *T. forsythia* | Success  Failure | 0.02 [0.01, 0.05]  0.02 [0.01, 0.03] | 0.01 [0.00, 0.03]  0.01 [0.01, 0.05] | 0.01 [0.01, 0.01]  0.01 [0.01, 0.02] |
| *T. denticola* | Success  Failure | 0.01 [0.01, 0.01]  0.01 [0.01, 0.02] | 0.01 [0.01, 0.01]  0.01 [0.01, 0.01] | 0.01 [0.01, 0.01]  0.01 [0.01, 0.01] |
| *C. rectus* | Success  Failure | 0.05 [0.04, 0.09]*, **  0.26 [0.17, 0.29] | 0.04 [0.04, 0.04]*, ***  0.04 [0.03, 0.04] | 0.12 [0.05, 0.19]**, ***  0.11 [0.09, 0.14] |
| *F. nucleatum* | Success  Failure | 1.94 [1.29, 5.10]  2.34 [1.73, 4.06] | 0.92 [0.00, 4.29]  0.49 [0.24, 3.68] | 3.08 [2.35, 6.92]  6.00 [4.83, 6.46] |
| *P. intermedia* | Success  Failure | 0.00 [0.00, 0.00]  0.00 [0.00, 0.00] | 0.00 [0.00, 0.01]  0.01 [0.00, 0.01] | 0.00 [0.00, 0.00]  0.00 [0.00, 0.00] |
| *P. nigrescens* | Success  Failure | 0.08 [0.07, 0.11]*  0.18 [0.11, 0.28] | 0.05 [0.04, 0.07]*, ***  0.04 [0.04, 0.15] | 0.12 [0.10, 0.18]***  0.04 [0.04, 0.07] |
| *A. actinomycetemcomitans* | Success  Failure | 0.00 [0.00, 0.00]  0.00 [0.00, 0.00] | 0.00 [0.00, 0.00]  0.01 [0.00, 0.00] | 0.00 [0.00, 0.00]  0.00 [0.00, 0.00] |
| *C. gingivalis* | Success  Failure | 0.05 [0.01, 0.25]  0.05 [0.04, 0.17] | 0.02 [0.01, 0.77]  0.35 [0.18, 0.45] | 0.03 [0.00, 0.09]  0.01 [0.01, 0.32] |
| *S. gordonii* | Success  Failure | 0.37 [0.33, 0.71]  0.55 [0.45, 0.71] | 0.28 [0.15, 0.61]  1.00 [0.75, 2.21] | 0.47 [0.21, 0.74]  0.29 [0.25, 0.54] |
| *S. intermedius* | Success  Failure | 0.03 [0.02, 0.04]*  0.18 [0.09, 0.28] | 0.00 [0.00, 0.00]*, ***  0.00 [0.00, 0.01] | 0.03 [0.02, 0.08]***  0.08 [0.05, 0.11] |
| *S. mutans* | Success  Failure | 0.08 [0.06, 0.12]*  0.08 [0.08, 0.09] | 0.00 [0.00, 0.00]*, ***  0.00 [0.00, 0.01] | 0.11 [0.06, 0.16]***  0.06 [0.05, 0.06] |

* *P* < 0.05 between baseline and date of treatment completion by Wilcoxon signed-rank test

** *P* < 0.05 between baseline and date of eradication (by the primary regimen) by Wilcoxon signed-rank test

*** *P* < 0.05 between date of treatment completion and determination of date of eradication (by the primary regimen) by Wilcoxon signed-rank test

1 week, date of treatment completion. 7 weeks, determination of date of eradication.
